# A prediction rule for lack of achievement of inactive disease with methotrexate as the sole disease-modifying antirheumatic therapy in juvenile idiopathic arthritis

**DOI:** 10.1186/s12969-019-0355-0

**Published:** 2019-07-25

**Authors:** Cecilia Bava, Federica Mongelli, Angela Pistorio, Marta Bertamino, Giulia Bracciolini, Sara Dalprà, Sergio Davì, Stefano Lanni, Valentina Muratore, Silvia Pederzoli, Silvia Rosina, Benedetta Schiappapietra, Chiara Suffia, Giulia Varnier, Sara Verazza, Gabriella Giancane, Alessandro Consolaro, Angelo Ravelli

**Affiliations:** 1 Università degli Studi di Genova, Dipartimento di Neuroscienze, Riabilitazione, Oftalmologia, Genetica e Scienze Materno-Infantili (DiNOGMI), Genova, Italy; 2 IRCCS Istituto Giannina Gaslini, Genova, Italy; 3 Azienda Ospedaliera Nazionale SS. Antonio e Biagio e Cesare Arrigo, Alessandria, Italy; 4 Fondazione IRCCS Ca’ Granda Ospedale Maggiore Policlinico, Milano, Italy; 5 IRCCS Fondazione Policlinico San Matteo, Pavia, Italy; 6 Ospedale Regina Montis Regalis, Mondovì, Italy; 7 Sechenov First Moscow State Medical University, Moscow, Russian Federation; 8 Clinica Pediatrica e Reumatologia, IRCCS Istituto Giannina Gaslini, Largo G. Gaslini 5, 16147 Genova, Italy

**Keywords:** Juvenile idiopathic arthritis, Pediatric rheumatology, Methotrexate, Predictors, Prediction rule, Biologic therapies

## Abstract

**Background:**

To investigate the frequency of achievement of inactive disease (ID) in children with juvenile idiopathic arthritis (JIA) treated with methotrexate (MTX) as the sole disease-modifyng antirheumatic (DMARD) therapy and to develop a prediction model for lack of attainment of ID.

**Methods:**

The clinical charts of consecutive patients started with MTX as the sole DMARD between 2000 and 2013 were reviewed. Patient follow-up was censored at first episode of ID or, in case ID was not reached, at last follow-up visit or when a biologic DMARD was prescribed. The characteristic at MTX start of patients who achieved or did not achieve ID were compared with univariate and multivariable analyses. Regression coefficients (β) of variables that entered the best-fitting logistic regression model were converted and summed to obtain a “prediction score” for lack of achievement of ID.

**Results:**

A total of 375 patients were included in the study. During MTX administration, 8.8% were given systemic corticosteroids and 44.1% intra-articular corticosteroids. After MTX start, 229 (61%) patients achieved ID after a median of 1.7 years, whereas 146 patients (39%) did not reach ID after a median of 1.2 years. On multivariable analysis, independent correlations with lack of achievement of ID were identified for the disease categories of systemic arthritis, enthesitis-related arthritis (ERA) and polyarthritis and C-reactive protein (CRP) >  1.4 mg/dl. The prediction score ranged from 0 to 3 and its cutoff that discriminated best between patients who achieved or did not achieve ID was > 0.5. The categories of systemic arthritis or ERA, both of which had a score greater than 0.5, were sufficient alone to predict a lower likelihood to reach ID. Polyarthritis and increased CRP, whose score was 0.5, assumed a predictive value only when present in association.

**Conclusion:**

A conventional treatment regimen based on MTX as the sole DMARD led to achievement of ID in a sizeable proportion of children with JIA. Our findings help to outline the characteristics of patients who may deserve a synthetic DMARD other than MTX or the introduction of a biologic DMARD from disease outset.

## Background

Juvenile idiopathic arthritis (JIA) is a chronic and heterogeneous disease characterized by prolonged synovial inflammation that may cause irreversible alterations of articular structures [1]. Joint changes can lead to serious impairment of physical function and have a major impact on the quality of life of children and their families [2–4]. It is now well established that minimizing disease activity over time reduces progression of joint damage and improves functional outcome in patients with chronic arthritis [5–7]. These observations, together with the recent therapeutic progress, have moved the therapeutic aims increasingly towards the attainment of an inactive disease status [8–10].

Methotrexate (MTX) is the cornerstone synthetic disease-modifying antirheumatic drug (DMARD) for the treatment of JIA [11–13]. It is an inexpensive and safe medication, and has been shown to be beneficial in 60–70% of patients both in randomized controlled trials and observational studies [14–17]. However, most analyses have evaluated the effectiveness of MTX in terms of percentage of improvement in clinical and laboratory indices of disease activity, whereas its capacity to induce complete disease quiescence has been seldom investigated [18–20]. Considering that current clinical practice mandates good overall disease control, to gain further insight into the therapeutic efficacy of MTX there is a need to obtain more information about its disease-remitting potential.

Because it is still impossible to predict the disease course in the individual patient and, hence, the treatment requirements at the onset of the disease [13, 21], a step-up approach is generally pursued of starting MTX and adding a biologic DMARD if the patient does not respond sufficiently well to MTX. However, given the abovementioned goal to start effective treatment immediately in order to prevent joint damage and the notion that MTX is not efficacious in a sizeable proportion of patients, it is essential to distinguish beforehand the patients who are likely to respond well to MTX from those who are not. The latter group may deserve prescription of a biologic DMARD from the outset. Over the years, several studies have sought for predictors of MTX efficacy. However, most of them have focused on the American College of Rheumatology (ACR) pediatric level of response (which emphasize percentage change), whereas the achievement of the state of inactive disease (ID) has rarely been used as endpoint [13, 22, 23].

Against this background, the primary aim of the present study was two-fold. First, we investigated the frequency of achievement of ID in children with JIA treated with MTX as the sole DMARD. Second, we aimed to develop a prediction model that could help to identify at treatment baseline the patients with a lesser likelihood to reach ID with MTX.

## Methods

### Study design and patient selection

All consecutive patients who met the International League of Associations for Rheumatology (ILAR) criteria for JIA [24], were started with MTX as the sole DMARD at the Istituto G. Gaslini of Genoa, Italy between 2000 and 2013, and had a minimum follow-up of 6 months after treatment initiation, were included in the study. Patients previously treated with any biologic DMARD were excluded. Previous treatment with other synthetic DMARDs or concomitant or previous administration of nonsteroidal anti-inflammatory drugs and systemic or intra-articular corticosteroids was allowed. The analysis was conducted through the retrospective review of patient clinical charts and data stored in clinical databases. Patient information was collected by means of standardized case report forms and was entered in a specialized database. The study protocol was approved by the ethics committee of the Istituto G. Gaslini, Genoa, Italy.

### Protocol of MTX administration

MTX was given orally or subcutaneously at the dosage of 10–15 mg/m2/week (maximum 25 mg/week) in a single weekly dose. All patients received folate supplementation with folinic acid at 25–50% of MTX dose the day after MTX administration. During MTX therapy, patients were evaluated clinically every 3 to 6 months. Laboratory monitoring was carried out every 8–12 weeks.

### Assessment of ID

For each patient, all visits from the start of MTX therapy to the last follow-up evaluation in which the patient was still receiving MTX as the sole DMARD were examined to verify whether the patient had achieved the state of ID. In case the attending physician had started to decrease the weekly dosage or space dosing further apart before the last follow-up visit because of achievement of ID, the last observation in which the patient was still receiving the weekly dose was considered as the last follow-up visit. In patients who achieved ID, the first visit in which ID was documented was retained.

The state of ID was defined, according to Wallace criteria [25], as no joint with active arthritis, no systemic manifestations attributable to JIA, no active uveitis, normal acute-phase reactants, and physician global assessment of overall disease activity indicating no disease activity (defined as score of 0 on a 0–10 visual analog scale). However, in a number of patients the full set of Wallace criteria could not be applied due to the lack of the physician global assessment of disease activity. For the visits in which this parameter was not available, but the other Wallace criteria were met, the absence of disease activity was inferred through the review of the patient chart by consensus of two investigators (CB and FM). To substantiate this judgement, the caring physician who originally examined the patient at the time of the visit was asked to review independently his/her notes and to confirm the inactivity of the disease. Disagreement between investigators and caring physician was resolved by consensus.

### Assessment of predictive factors

Variables recorded at the time of MTX start comprised sex, age at disease onset, age and disease duration, ILAR category, antinuclear antibody (ANA) status, route of MTX administration, active joint count, erythrocyte sedimentation rate (ESR), and C-reactive protein (CRP). Predictive variables also included therapeutic interventions made before MTX start and concomitant therapies during MTX administration. Patient follow-up was censored at the time of first occurrence of ID or, in case ID was not achieved, at last follow-up visit or at the time when a biologic DMARD was prescribed.

### Statistics

Descriptive statistics were reported as medians and interquartile ranges for continuous variables and as absolute frequencies and percentages for categorical variables. Comparisons between patients who did or did not achieve ID were performed by Mann-Whitney U test in case of quantitative data and chi-square test or Fisher’s exact test, as appropriate, for categorical data.

Predictive factors were tested for association with lack ofachievement of ID during the time of observation through multiple logistic regression analysis, entering explanatory variables that showed statistically (*p* < 0.05) significant results in univariate tests or were considered clinically meaningful. Cases with missing variables were excluded from the analysis. Before the application of logistic regression procedures, continuous variables, including active joint count, ESR and CRP, were dichotomized to binary variables. Cut-off points were obtained through receiver operating characteristic (ROC) curve analysis. The step-down strategy of analysis was chosen, which consists of examining the effect of removing variables from the saturated model.

To obtain a “prediction model” of lack of achievement of ID, the regression coefficients (β) of predictive variables that entered the best-fitting logistic regression model were converted into scores rounded to the nearest 0.5 and then summed up to obtain a “prediction score”. Finally, by means of the ROC curve method, the cutoff score that discriminated best between patients who achieved or did not achieve ID was calculated.

The statistical packages used were Statistica (version 9.0, StatSoft Corp.) for univariate analyses and Stata (release 7, Stata Corp.) for multivariable analyses.

## Results

### Patient characteristics

A total of 406 patients were treated with MTX as the sole DMARD in the study period. Thirty-one patients were excluded from the analysis because the clinical chart could not be retrieved or the follow-up period after start of MTX was shorter than 6 months. None of the patients had died due to disease complications, particularly macrophage activation syndrome. The main demographic and clinical features of the remaining 375 patients are presented in Table 1. The patient sample was characterized by marked prevalence of females, young age at disease onset, and high frequency of positive ANA status. The most common ILAR categories were oligoarthritis (44.3%) and RF-negative polyarthritis (37.1%), followed by systemic arthritis (7.7%), enthesitis-related arthritis (ERA) (5.1%), undifferentiated arthritis (3.7%), RF-positive polyarthritis (1.9%), and psoriatic arthritis (0.3%). Of the 166 patients with oligoarthritis, 24% had persistent oligoarthritis and 20.3% had extended oligoarthritis.

**Table 1:** Baseline characteristics of study patients considered as a whole and by achievement of ID

Features	All patients (*n* = 375)	Patients who did not achieve ID (*n* = 146)	Patients who achieved ID (*n* = 229)	*P*^#^
Gender				0.0004
Female	299 (79.7)	103 (70.5)	195 (85.6)	
Male	76 (20.3)	43 (29.5)	33 (14.4)	
Median (IQR) age at disease onset, yrs	3.2 (1.7–7.0)	4.4 (1.9–8.2)	2.7 (1.7–5.5)	0.002
Median (IQR) age, yrs	5.6 (3.0–9.5)	6.3 (3.3–9.9)	4.9 (2.8–9)	0.05
Median (IQR) disease duration, yrs	0.8 (0.4–2.4)	0.8 (0.3–2.5)	0.9 (0.4–2.4)	0.08
Median (IQR) follow-up time, yrs	1.5 (0.8–2.5)	1.2 (0.5–2.6)	1.7 (1–2.5)	0.002
Functional phenotypes^§^				< 0.0001
Systemic arthritis	29/373 (7.8)	25 (17.2)	4 (1.8)	
Polyarthritis	151/373 (40.5)	59 (40.7)	92 (40.3)	
Oligoarthritis	174/373 (46.6)	49 (33.8)	125 (54.8)	
Enthesitis-related arthritis	19/373 (5.1)	12 (8.3)	7 (3.1)	
Patients with positive ANA	264/371 (71.2)	79/133 (59.4)	185/226 (81.9)	< 0.0001
Median (IQR) no. of active joints	5 (3–8)	5 (3–10)	5 (3–7)	0.51
Median (IQR) ESR, mm/h (*n* = 313)	40 (21–58)	46 (24–62)	35.5 (19–56)	0.02
Median (IQR) CRP, mg/dl (*n* = 315)	1.3 (0.5–3.6)	2 (0.5–4.4)	0.9 (0.5–3)	0.001

*ID* inactive disease, *IQR* interquartile range, *ANA* antinuclear antibodies, *ESR* erythrocyte sedimentation rate, *CRP* C reactive protein

^#^*P* value refers to the comparison between patients who did not achieve or achieved ID. ^§^For the purposes of the study analyses, the ILAR categories of juvenile idiopathic arthritis were grouped in functional phenotypes according to Beukelman et al. (ref. [26])

Data are the number (%) unless otherwise indicated

For the purpose of the analysis and according to Beukelman et al. [26], patients were grouped in the functional phenotypes of oligoarthritis (4 or fewer affected joints), polyarthritis (5 or more affected joints), systemic arthritis, and ERA. At treatment start, patients had on average early disease, as shown by the median disease duration of 0.8 years and the median duration of follow-up at our center of 0.2 months. Twenty-one of the 29 patients with systemic arthritis had active systemic manifestations. The median number of affected joints was 5 and the most frequently involved joints were the ankle and the knee.

The MTX regimen, the therapeutic interventions made before MTX start and the medications administered concomitantly at treatment start or during MTX therapy are shown in Table 2. MTX was started more commonly subcutaneously than through the oral route (55.9 vs 44.1%). Nearly half of the patients who were started orally were subsequently switched to the parenteral route, most frequently due to insufficient efficacy. Only a few patients had received corticosteroids, either systemic or intra-articular, or other synthetic DMARDs before the start of MTX. At MTX initiation, nearly two third of the patients were receiving NSAIDs, 21.8% were taking systemic corticosteroids, and around 40% were given intra-articular corticosteroids. During MTX administration, approximately 9% of the patients were prescribed systemic corticosteroids and 44.1% underwent intra-articular corticosteroid injections. The systemic corticosteroid medication was prednisone in almost all patients, whereas the corticosteroid preparation used for intra-articular injections was triamcinolone hexacetonide for large joints and methylprednisolone acetate for small joints or for joints difficult to access clinically (e.g. the subtalar and intertarsal joints) [27].

**Table 2:** Therapeutic data of study patients considered as a whole and by achievement of ID

Therapeutic features	All patients (*n* = 375)	Patients who did not achieve ID (*n* = 146)	Patients who achieved ID (*n* = 229)	*P*^#^
Median MTX dose, mg/m^2^ (*n* = 339)	12.8 (11.1–14.5)	13.1 (11.6–14.5)	12.8 (10.9–14.3)	0.2
Route of MTX administration				0.04
Oral	162/367 (44.1)	53 (37.3)	109 (48.4)	
Parenteral	205/367 (55.9)	89 (62.7)	116 (51.6)	
Treatment before MTX start				
Intra-articular corticosteroid injections	17/372 (4.6)	3/143 (2.1)	14 (6.1)	0.07
Systemic corticosteroids	29/372 (7.8)	22/143 (15.4)	7 (3.1)	< 0.0001
Other synthetic DMARDs	21 (5.6)	14/143 (9.8)	7 (3.1)	0.006
Concomitant therapies during MTX administration				
Intra-articular corticosteroid injections	165/374 (44.1)	67/145 (46.2)	98 (42.8)	0.52
Systemic corticosteroids	33/374 (8.8)	25/145 (17.2)	8 (3.5)	< 0.0001

*ID* inactive disease, *MTX* methotrexate, *DMARDs* disease modifying antirheumatic drugs

^#^*P* value refers to the comparison between patients who did not achieve or achieved ID

Data are the number (%) unless otherwise indicated

### Frequency of achievement of ID

A total of 229 (61%) patients achieved the state of ID after a median of 1.7 years (IQR 1–2.5 years) from the start of MTX, whereas 146 patients (39%) did not achieve ID after a median of 1.2 years (IQR 0.5–2.6 years) of MTX therapy. In 48 (20.9%) of the 229 patients with ID, this state could not be established formally according Wallace criteria, owing to the lack of the physician global assessment, but was inferred through the review of clinical charts. This assessment was made by the caring physicians for all 48 patients.

### Comparison of the clinical characteristics between patients with and without ID

The demographic, clinical, laboratory and therapeutic features of patients who achieved or did not achieve ID are reported in Tables 1 and 2. Compared with patients who reached ID, those who did not were less frequently females, were older at disease onset, had less frequently oligoarthritis and more frequently ERA and systemic arthritis, were less frequently ANA positive, were given more commonly parenteral MTX, and had higher ESR and CRP values. In addition, patients who did not attain ID had received more frequently systemic corticosteroids either before MTX start or during MTX administration. There was no difference between the two groups in age and disease duration at study entry, number of active joints, and frequency of intra-articular corticosteroid therapy either before MTX start or during MTX therapy.

### Results of multivariable analysis

For the multivariable analysis, complete data were available for 369 patients. However, 4 children with ERA and axial disease were excluded from the analysis based on the demonstrations in the adult literature that traditional DMARDs are ineffective in the management of axial spondyloarthritis. In addition, because the administration of corticosteroid therapy could simply reflect the provider perception that the child had severe disease at onset and did not constitute a disease characteristic, this variable was not included in multivariable analysis. The best-fitting model obtained through logistic regression procedures, in which the lack of attainment of ID was the dependent variable, is presented in Table 3. Independent correlations with lack of achievement of ID were identified for the functional categories of systemic arthritis, ERA and polyarthritis (versus oligoarthritis) and a CRP value greater than 1.4 mg/dl.

**Table 3:** Regression logistic model for the lack of achievement of ID

	β	OR (95% CI)	*P*^a^	Score
Functional phenotype (*reference phenotype: oligoarthritis*)			< 0.0001	
Polyarthritis	0.26	1.3 (0.8–2.1)		0.5
Enthesitis-related arthritis	1.37	3.9 (1.3–11.8)		1.5
Systemic arthritis	2.45	11.6 (3.7–36.0)		2.5
CRP > 1.4 mg/dl (*reference category: ≤ 1.4 mg/dl*)	0.60	1.8 (1.1–2.9)	0.014	0.5
Score range				0–3

*ID* inactive disease, *OR* Odds Ratio, *95% CI* 95% Confidence Interval; ^a^Likelihood Ratio Test; *CRP* C reactive protein

Complete data were available for 365 patients. This model was transformed, using the β regression coefficient, into a prediction score for the risk of lack of achievement of ID. The area under the curve (AUC) of the model was 0.67.

### Development of the prediction model

The score assigned to each variable independently associated with the lack of achievement of ID in multivariable analysis is shown in Table 3. Note that the score of each functional phenotype is mutually exclusive. The prediction score obtained though the sum of the individual scores ranged from 0 to 3. Sensitivity and specificity were calculated for several cutoffs of the prediction score, as shown in Table 4. The cutoff score that discriminated best between patients who achieved or did not achieve ID was > 0.5. Its sensitivity and specificity were 54.8 and 66.4%, respectively. The assessment of accuracy through the ROC curve analysis yielded an area under the curve (AUC) of 0.64.

**Table 4:** Sensitivity and specificity for each score cutoff

Prediction score	Sensitivity (%)	Specificity (%)
= 0	100	0
> 0	86.3	21.8
> 0.5	54.8	66.4
> 1	28.8	90.8
> 1.5	23.3	95.6
> 2	17.1	98.3
> 2.5	11.6	98.7

Area under ROC curve: 0.64; 95% confidence interval: 0.59–0.69

The best score cut-off value is > 0.5. A score > 0.5 had the best capacity to identify patients who did not reach inactive disease

## Discussion

In the present study, we investigated the frequency of attainment of ID in 375 children with JIA seen between 2000 and 2013 who received MTX as the sole DMARD, with or without concomitant administration of NSAIDs or systemic or intra-articular corticosteroids. Of the two routes of corticosteroid administration, the intra-articular one likely played a greater synergistic role with MTX as 44.1% of patients were given corticosteroids intra-articularly and only 8.8% systemically during MTX administration. Patient follow-up was censored at the time of the occurrence of the first episode of ID, at last follow-up visit with persistently active disease, or when the caring physician deemed necessary the start of a biologic DMARD because of persistently active disease despite MTX therapy. Thus, the results of our study provide insights on the potential to achieve complete disease quiescence with conventional (i.e. non-biologic) treatment. As such, they offer a measure against which outcomes from other cohorts may be judged and a benchmarking for outcome comparisons with recent cohorts treated with more aggressive approaches, based on earlier introduction of biologic DMARDs or with the treat-to-target strategy [9, 28, 29].

We found that 61% of patients achieved ID after a median of 1.7 years from the start of MTX therapy. The relative frequency of favorable outcome was higher among patients with oligoarthritis than in those with ERA or systemic arthritis; an equal proportion of patients with polyarthritis reached or did not reach ID. Note that in the study period it was our policy to inject with corticosteroids all active joints at presentations in each patient with oligoarthritis. MTX was started at the time of intra-articular therapy in most of these patients, except those with knee monoarthritis who received their first injection [30]. The results of our study are not easily comparable with those reported in other series of JIA patients treated with MTX because of differences in proportion of disease categories, length of follow-up, treatment regimens, concomitant therapies and outcome endpoints. In a recent German national multicenter study, a similar proportion (68%) of biologic-naïve patients who were started with MTX experienced at least one episode of ID [23].

In univariate analysis, patients of male gender and with older age at disease onset, ERA or systemic arthritis, absence of ANA, and higher ESR and CRP, and patients treated with parenteral MTX and given systemic corticosteroids before and during MTX administration were less likely to attain ID. The lesser responsiveness to MTX of patients with systemic arthritis is in keeping with the well-established notion that this JIA category is scarcely susceptible to this drug, particularly in the presence of active systemic manifestations [31]. MTX is also known to be distinctively less effective in ERA, for which current therapeutic recommendations advice the use of sulfasalazine as primary synthetic DMARD [26]. The predictive role of male gender and older onset age is likely related to that of systemic arthritis and ERA, in which these features are more prevalent than in the other forms of JIA. The relationship between absence of ANA and poorer efficacy of MTX agrees with the previous demonstrations of ANA-positivity being a marker of better response to MTX [13, 32–35]. Elevated acute phase reactants were found by Bulatovìc et al. [35] to be associated with lack of achievement of ID with MTX therapy. The association of parenteral MTX with worse outcome may reflect our policy to switch from the oral to the parenteral route or to start MTX parenterally in the most severe or refractory patients. Likewise, patients treated with systemic corticosteroids were those with systemic arthritis or more severe polyarthritis, who have an underlying high risk of poorer therapeutic response.

The variables that remained independently associated with lack of attainment of ID in the best-fitted model of logistic regression procedures were systemic arthritis, ERA, polyarthritis and elevated CRP. Based on the results of multivariable analysis, we devised a prediction score for lack of achievement of ID with MTX as the sole DMARD, which ranged from 0 to 3. The score cutoff that discriminated best between patients who achieved or did not achieve ID was > 0.5, which means that patients with a score ≥ 1 are less likely to attain ID with MTX as the sole DMARD. Thus, a diagnosis of systemic arthritis or ERA, both of which had a score greater than 0.5, was sufficient alone to predict a lower likelihood to reach ID. Polyarthritis and increased CRP, whose score was 0.5, assumed a predictive value only when present in association.

A number of caveats should be considered in interpreting our findings. The study design was retrospective, which implies the risk of missing or possibly erroneous data. Our results reflect a single-center experience, which means that they may not be generalized to series followed in other settings. Because our analysis was nonrandomized and observational, we cannot exclude that patients who achieved ID had a less aggressive disease than those who did not. In this respect, the overrepresentation of the oligoarticular phenotype, which is regarded as the most benign JIA subset, might partly explain the favorable outcome figures. The median time interval of 1.7 years between MTX start and achievement of ID would nowadays be regarded as too long. Contemporary treatment strategies mandate an earlier achievement of complete disease control [9, 10]. We recognize that the AUC of the predictive model as well as the sensitivity and specificity of the score cutoff were only fair. Thus, the prediction model needs to be tested in a validation cohort prospectively. The use of logistic regression cannot account for the different length of follow-up time for each patient. A time-to-event hazard model would have been more appropriate to address this issue. Achievement of ID was only assessed at a single point in time and not in terms of time spent in ID. Several studies have shown a high risk of disease flare after MTX discontinuation for clinical remission in children with JIA [20, 23, 36]. We should finally recognize that in a number of patients the state of ID could not be established formally according Wallace criteria, owing to the lack of the physician global assessment, but was inferred through the review of clinical charts.

## Conclusions

In conclusion, we found that a conventional treatment regimen based on the use of MTX as the sole DMARD led to the achievement of ID in a sizeable proportion of children with JIA. Patients with systemic arthritis, ERA and polyarthritis with increased CRP were less likely to achieve ID. These findings help to outline the characteristics of JIA patients who may deserve the prescription of a synthetic DMARD other than MTX or the introduction of a biologic DMARD from the disease outset.

## Data Availability

The datasets used in the study are available from the corresponding author upon request.

## Abbreviations

ACR: American College of Rheumatology
ANA: Antinuclear antibody
AUC: Area under the curve
CRP: C-reactive protein
DMARD: Disease-modifying antirheumatic drug
ERA: Enthesitis-related arthritis
ESR: Erythrocyte sedimentation rate
ID: Inactive disease
ILAR: International League of Associations for Rheumatology
JIA: Juvenile idiopathic arthritis
MTX: Methotrexate
ROC: Receiver operating characteristic
